# Transcriptome Analysis of Maternal Gene Transcripts in Unfertilized Eggs of *Misgurnus anguillicaudatus* and Identification of Immune-Related Maternal Genes

**DOI:** 10.3390/ijms21113872

**Published:** 2020-05-29

**Authors:** Chan-Hee Kim, Eun Jeong Kim, Chaehwa Seo, Yoon Kwon Nam

**Affiliations:** 1 Department of Marine Bio-Materials and Aquaculture, College of Fisheries Sciences, Pukyong National University, 45 Yongso-ro, Nam-gu, Busan 48513, Korea; chkim@pknu.ac.kr (C.-H.K.); kejung03@naver.com (E.J.K.); 2 Bioinformatics Team, DNA Link Inc., 150 Bugahyeon-ro, Seodaemun-gu, Seoul 03759, Korea; chseo@dnalink.com

**Keywords:** de novo transcriptome, maternal gene transcript, unfertilized egg, immune-related gene, *Misgurnus anguillicaudatus*

## Abstract

Maternal genes are important in directing early development and determining egg quality in fish. We here report the de novo transcriptome from four tissue libraries of the cyprinid loach, *Misgurnus anguillicaudatus*, and for the first time identified maternal gene transcripts in unfertilized eggs and suggest their immune system involvement. Expression profiles and functional enrichment revealed a total 24,116 transcripts were expressed as maternal transcripts in unfertilized eggs, which were involved in a wide range of biological functions and pathways. Comparison expression profiles and analysis of tissue specificity revealed that the large numbers of maternal transcripts were stored in unfertilized eggs near the late phase of ovarian maturation and before ovulation. Functional classification showed a total of 279 maternal immune-related transcripts classified with immune system process GO term and immune system KEGG pathway. qPCR analysis showed that transcript levels of identified maternal immune-related candidate genes were dynamically modulated during development and early ontogeny of *M. anguillicaudatus*. Taken together, this study could not only provide knowledge on the protective roles of maternal immune-related genes during early life stage of *M. anguillicaudatus* but could also be a valuable transcriptomic/genomic resource for further analysis of maternally provisioned genes in *M. anguillicaudatus* and other related teleost fishes.

## 1. Introduction

Before embryos begin to utilize newly synthesized products derived from the zygotic genome, maternally inherited components such as RNA transcripts and proteins direct virtually all aspects of early development in animal embryos [1,2,3,4]. Maternal components that are stored into an egg during oogenesis are particularly important in most oviparous teleost fish, as they undergo external fertilization and development, without close interconnection with their parents [5,6,7]. Besides, it was proposed that maternal components are a critical determinant of fish egg quality, which serves as a key challenging issue in the reproductive control of aquacultured fish [8,9,10,11,12].

Fish embryos and early larvae are often exposed to various potential pathogens in the environment. Accordingly, a need to protect themselves from pathogenic invaders is crucial during this early life interval. The immune system in vertebrates including teleost fishes utilizes complex and highly organized gene sets of molecular signaling pathways that protect against environmental invasions and regulate dynamic changes in an organism [13,14]. This complex and organized immune system would make to intertwine and share certain common features in developmental mechanisms and operational modes. It is not surprising that many immune-related genes co-opt onto the developmental mechanisms of animals. For an instance, the membrane receptor Toll and the related Toll-like receptors (TLR) are well known for their function in innate immunity, however, these molecules were originally found in studies investigating embryonic development [15,16]. Maternal immunity is believed to be the principal means of fish embryo and larval protection until they have developed a complete immune system [5]. It is of interest, therefore, to identify maternal immune-related genes/transcripts to extend insights into dynamic changes during the early embryonic development process. To date, most studies on the maternal immunity of fish have mainly focused on immune effector molecules such as peptides/proteins: antimicrobial peptides, immunoglobulins/antibodies, complement components, lectins, and lysozyme [5,17,18]. However, in contrast to the rather rich studies on immune factors, a study that builds a comprehensive understanding of maternal immune gene transcripts in fish species remains limited. Recently, next-generation sequencing (NGS) technologies have revolutionized our ability to analyze the dynamic gene transcripts in a specific cell, tissue, or organism at a given developmental stage [11,19,20,21]. As the analysis of massive quantities of genes has allowed gene discovery and expression profiling, recent transcriptome data for several fishes have provided notable insights into the distinctive features in oocyte development and early embryonic development [19,22,23,24,25].

The cyprinid loach, *Misgurnus anguillicaudatus* (Actinopterygii; Cypriniformes; and Cobitidae), also known as oriental weather loach, is widely distributed in freshwater areas. *M. anguillicaudatus* prefers a habitat with muddy bottoms as a spawning ground, which is known to contain abundant and diverse microbial populations. The loach has been considered a valuable source of food owing to its high nutritional values in East Asia including Korea and, has become an important freshwater aquaculture species (https://www.fishbase.de/summary/Misgurnus-anguillicaudatus.html). Moreover, this species has many advantageous merits as an experimental model organism, particularly with respect to developmental and reproductive researches. They include short generation time, year-round multiple spawning under controlled laboratory conditions, high fecundity, and fast and transparent embryonic development [26,27,28,29]. These attractive characteristics make this fish a suitable model organism to study the contribution of maternally provisioned immune-related gene transcripts to development and host defense function in the early life stages of teleosts.

Here, we report the *M. anguillicaudatus* transcriptome newly constructed from RNA-Seq libraries derived from unfertilized eggs, ovary, testis, and muscle tissues. We employed a paired-end Illumina HiSeq 2500 sequencing technology and the subsequent de novo assembly, to generate a comprehensive set of reference transcripts. The transcriptome assembly was used to analyze expression profiles and identify maternal gene transcripts related to immune functions. Further, we analyzed transcriptional levels of identified maternal immune-related candidate genes during the development of *M. anguillicaudatus*. This study is the first transcriptome analysis of maternal gene transcripts associated with immune function in the loach, *M. anguillicaudatus*.

## 2. Results and Discussion

### 2.1. Sequencing, De Novo Assembly, and Completeness

The RNA-Seq libraries from muscle, testis, ovary, and unfertilized eggs were constructed to carry out paired-end sequencing using the Illumina HiSeq 2500 platform. The statistics for the sequencing and de novo assembly are shown in Table 1. Illumina sequencing produced a total of 243,267,764 raw reads from the four different cDNA libraries. The maximum number of raw reads generated from the ovary library and the minimum number of raw reads generated from the muscle library were 63,490,556 and 56,211,235, respectively. After trimming adaptor sequences and ambiguous and low-quality reads via quality filtering, a total of 237,869,744 clean reads (approximately 118 million paired-end clean reads) were obtained using the Trimmomatic software [30]. Trinity assembler was then used to pool and assemble the clean reads into 281,866 contigs, with a total length of 218,148,233 bp [31]. Redundant sequences were removed from the assembled contigs using the CD-HITest platform [32]. This yielded a total of 267,111 non-redundant transcripts with a total length of 197,693,642 bp, a mean length of 740.12 bp, weighted median length (N50) of 1533 bp, and a GC (guanine+cytosine) ratio of 42.90% in a size range of 201–23,427 bp. Although the N50 statistic is not entirely appropriate for de novo transcriptome assemblies, it is still a traditionally valuable measurement for the assessment of continuity in transcriptome assembly [33,34].

Thus, the N50 value (1533 bp) of our transcriptome assembly was comparable to those obtained for published de novo transcriptomes of *M. anguillicaudatus* [21,35,36,37]. The detailed length distribution of de novo assembled transcriptome is shown in Figure S1. Of these non-redundant transcripts, the majority of sequences (157,612, 60.0%) ranged from 200 to 400 bp, 55,464 (20.8%) from 400 to 800 bp, and 49,802 (18.6 %) were longer than 1 kb. Completeness of the assembly was evaluated using BUSCO against the eukaryote (eukaryote_odb9) lineages [38,39], which revealed that 100% of conserved genes across all eukaryotes were present; 70.6% complete and single-copy BUSCOs (214 out of 303) and 29.4% complete and duplicated BUSCOs (89 out of 303). Taken together with the sufficient number of sequencing clean reads mentioned above, the transcriptome assembly from the four cDNA libraries of the loach *M. anguillicaudatus* achieved a reasonable degree of completion and an acceptable coverage and quality of protein-coding transcripts for subsequent analysis.

### 2.2. Functional Annotation of the Reference Transcriptome

**Figure 1:**
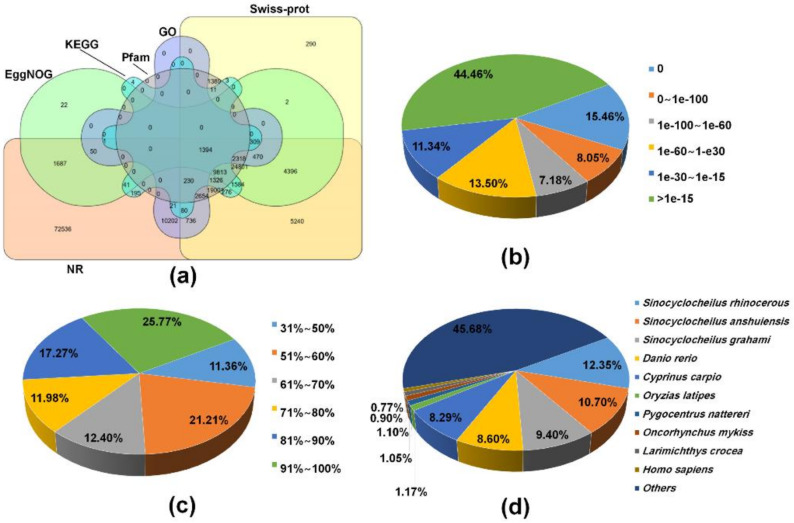
Functional annotation of transcripts from the de novo assembled *M. anguillicaudatus* transcriptome: (**a**) Venn diagram of all annotated transcripts from the reference transcriptome against non-redundant (NR), Swiss-Prot, Pfam, non-supervised orthologous groups (eggNOG), gene ontology (GO), and Kyoto Encyclopedia of Genes and Genomes (KEGG) databases; (**b**) E-value distribution; (**c**) similarity distribution; and (**d**) species distribution.

To find homologous proteins and the collection of biological information from various sources, all of 267,111 transcripts were used to search homology against several public databases based on the BLAST algorithm. Overall, 161,091 (60.3%) transcripts were successfully annotated to a least one database and 1394 (0.5%) transcripts shared annotation in all databases used (Figure 1a). Of the annotated transcripts, 159,361 (98.9%), 76,332 (47.4%), 62,946 (39.1%), 46,897 (29.1%), 18,465 (11.5%), and 15,288 (9.5%) of transcripts had BLAST hit to NR, Swiss-Prot/UniproKB, EggNOG, HMMER/Pfam, GO, and KEGG databases, respectively (Figure 1a and Table S1). Moreover, a total of 70,203 ORFs were predicted from 64,767 (24.2%) transcripts using TransDecoder, of which 4,481 (6.4%) and 12,597 (17.9%) ORFs contained a signal peptide and transmembrane region by SignalP and tmHMM, respectively. Meanwhile, remaining sequences (106,020, 39.7%) resulted in non-significant hits. This can be explained by a lack of sequences conservation across species associated with incomplete gene information on non-model species in public databases, or because it contains non-coding RNA in sequences of the transcripts. Alternatively, short length transcripts obtained through de novo assembly would reduce the BLAST annotation efficiency. The e-value distribution of the transcripts in the NR BLAST results revealed that 48,916 (30.69%) annotated transcripts showed significant homology (less than 1e-60) and 70,848 (44.46%) transcripts were annotated with e-value of greater than 1e-15 (Figure 1b). The ratio of the similarity distributions revealed 67.43% of the NR annotated transcripts had a similarity over 61% (Figure 1c). The species distribution analysis showed that 159,361 transcripts were distributed in 7,871 species. The top-hit species belonged to the genus *Sinocyclocheilus*, which include *S. rhinocerous* (19,676, 12.3%), *S. anshuiensis* (17,053, 10.7%), and *S. grahami* (14,974, 9.4%). Then, these species were followed by *Danio rerio* (13,709, 8.6%), and Cyprinus carpio (13,206, 8.3%; Figure 1d). These species belong to the Cyprinidae family along with *M. anguillicaudatus*, and their annotated genomes are available in a database (https://www.ncbi.nlm.nih.gov/genome/annotation_euk/). In contrast, a previous study reported that transcripts of *M. anguillicaudatus* skin transcriptome showed relatively high homology to *D. rerio* [37]. This may be ascribed to several reasons including the relatively small number of transcripts obtained from one specific tissue and a restricted use of the database (i.e., NR protein and *D. rerio* transcriptome database) [37].

**Figure 2:**
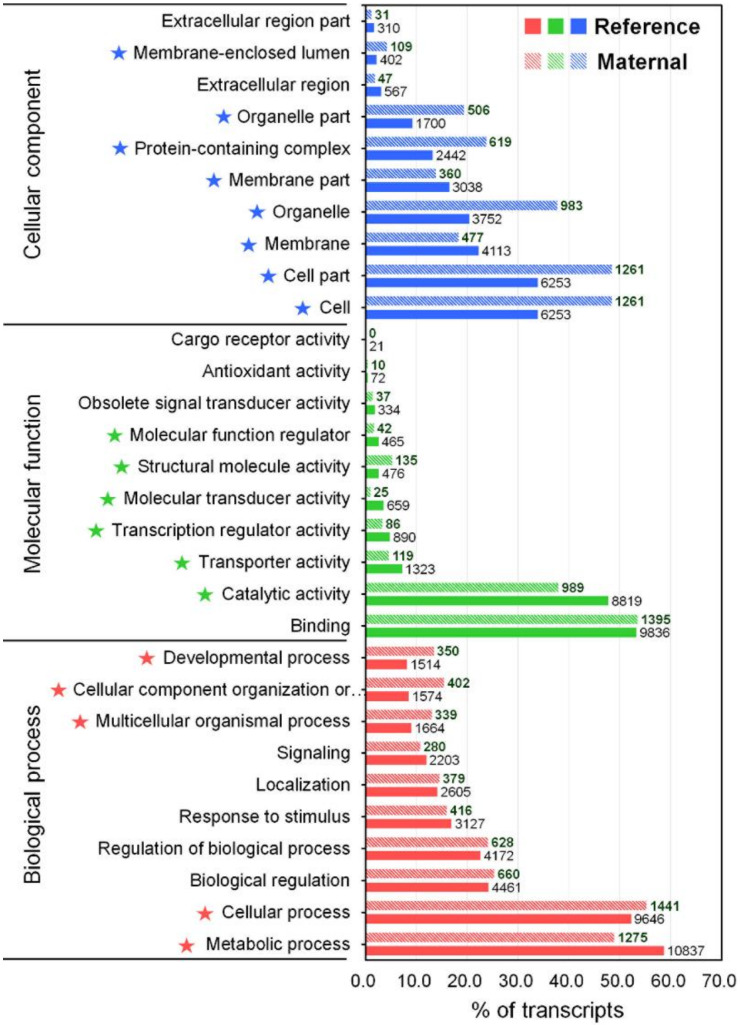
GO classification for the reference transcriptome and enrichment analysis of maternal gene transcripts using the Pearson Chi-Square test. Horizontal axis displays the percentage of significant transcripts in each column of which the number at the end indicates the number of transcripts. Vertical axis displays the detailed GO term corresponding to each functional type. Star marks in front of terms indicate significantly enriched GO terms (*p* < 0.05).

The GO annotation is a standardized system for functional classification and inference of the biological significance of genomic/transcriptomic datasets [40]. Therefore, the NR BLAST results were submitted to the Blast2GO program [41], yielding 18,465 (11.6%) transcripts that were categorized into a total 92,581 GO terms because of the large numbers of transcripts annotated with more than one GO term (Table S2). The GO annotation result was represented by three main categories at level 2 including biological process (BP, 50 subcategories), molecular function (MF, 23 subcategories), and cellular component (CC, 21 subcategories) using the WEGO tool (Figure 2 and Table S2) [42]. In the BP category, the most represented GO terms were the metabolic process (10,837, GO:0008152), cellular process (9646, GO:0009987), biological regulation (4461, GO:0065007), regulation of biological process (4461, GO:0050789), and response to stimulus (3127, GO:0050896). The immune system process (GO:0002376) in BP contained 418 transcripts. For the MF category, transcripts were mainly associated to binding (9836, GO:0005488), and catalytic activity (8819, GO:0003824). In the CC category, cell (6253, GO:0005623), cell part (6253, GO:0044464), membrane (4113, GO:0016020), organelles (3752, GO:0043226), and membrane part (3038, GO:0044425) were the most represented categories. Direct comparisons between the number of transcripts in each GO category of our transcriptome assembly and previously published transcriptomic data for *M. anguillicaudatus* are somewhat limited. Nevertheless, the representation of GO distribution was consistent between our transcriptome and previous transcriptome assemblies for all three main categories [35,36,37,43].

**Figure 3:**
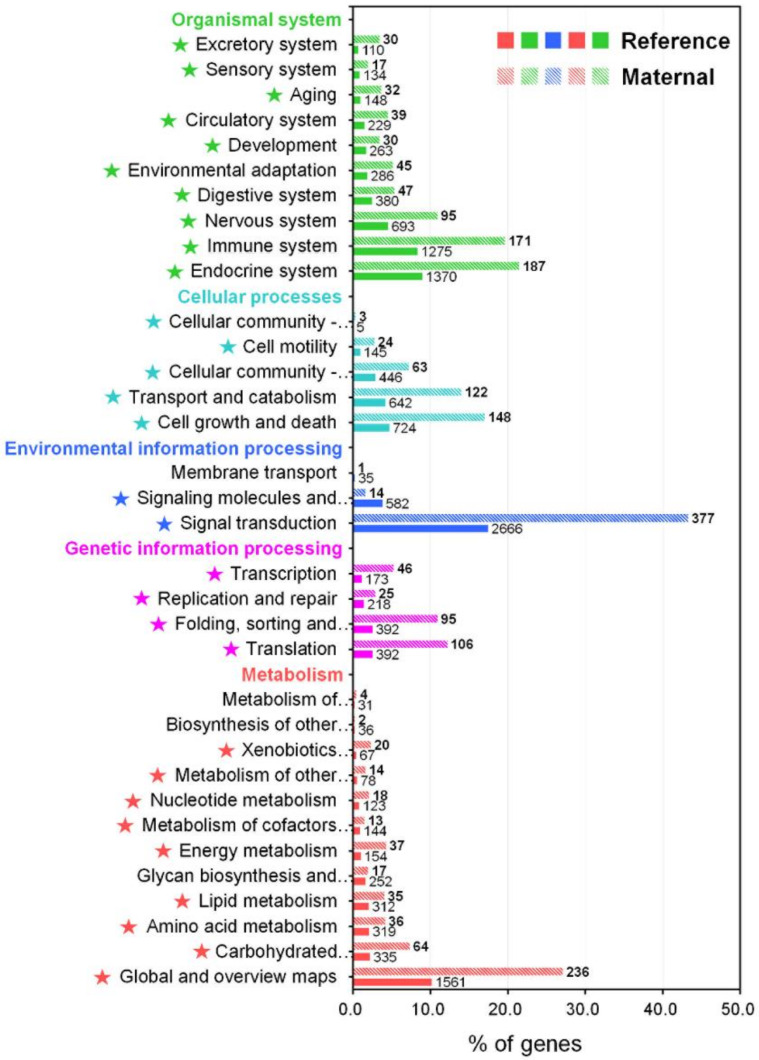
KEGG pathway annotation for the reference transcriptome and enrichment analysis of maternal gene transcripts using the Pearson Chi-Square test. Star marks in front of pathways indicate significantly enriched KEGG pathway (*p* < 0.05).

The KEGG pathway provides an important insight into different biological processes and interactions across different tissues or conditions within an animal, enabling further understanding of the biological function and interaction of the transcripts in *M. anguillicaudatus* [44,45]. Consequently, 15,288 transcripts (8.4%) were assigned to 4413 unique KEGG orthology (KO) identifiers and grouped into 402 KEGG signaling pathways (Table S2). The annotated pathways were represented in five main categories: metabolism (3412 KOs), genetic information processing (1175 KOs), environmental information processing (3283 KOs), cellular processes (1962 KOs), and organismal systems (4888 KOs; Figure 3 and Table S2). In these main categories, the signal transduction pathways had the largest number of KOs (2666), followed by global and overview maps (1561), endocrine system (1370), and immune system (1275). These functional classifications could provide comprehensive information for the understanding of the biological significance and function of transcripts in the *M. anguillicaudatus* transcriptome.

### 2.3. Expression Analysis and Characterization of Maternal Transcripts

**Figure 4:**
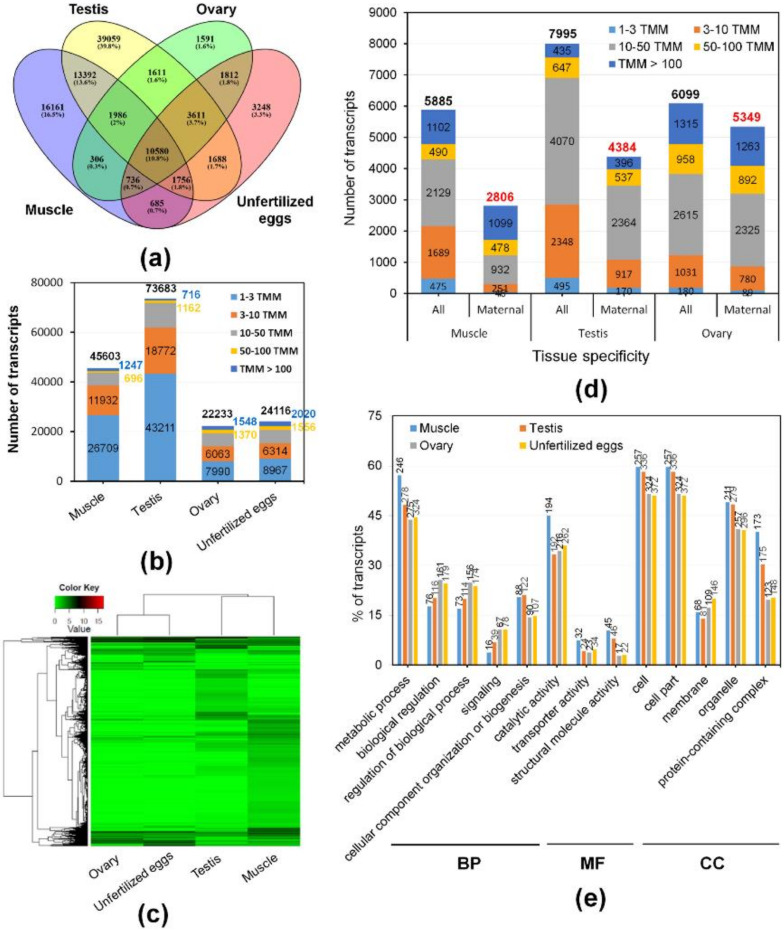
Expression analysis of transcripts in different tissues of *M. anguillicaudatus* and characterization of maternal transcripts: (**a**) overlapping comparison of expressed transcripts with a cut-off trimmed mean normalization of M-values (TMM) value < 1 in each tissue; (**b**) the number of transcripts with different expression abundances in each tissue; (**c**) heatmap of maternal transcripts with substantial expression differences across tissues; and (**d**) the number and abundance of transcripts with tissue-specificity by the TissueEnrich tool, which were coexpressed in more than two tissues. “Maternal” in the horizontal axis represented transcripts that were coexpressed in the unfertilized eggs with other tissues; (**e**) GO terms (*p* < 0.05) that contained significantly different numbers of maternal transcript with tissue-specificity.

To gain insight into the composition and complexity of transcripts that were particularly expressed in the transcriptome of *M. anguillicaudatus* unfertilized eggs, we evaluated the expression levels and classified the transcripts from each RNA-seq library. Overall, more than 89% of transcript sequences for each library were mapped to the reference transcriptome assembly, of which 95% of transcript sequences in unfertilized eggs were mapped (see Table 1 mapping ratio). Expression levels of transcripts in each sample were calculated and shown in Table S3. With a cut-off value of 1 trimmed mean normalization of M-values (TMM), a total of 98,222 (36.8%) transcripts were detected in at least one tissue, with 45,603 (46.4%) in muscle, 73,683 (75.0%) in testis, 22,233 (22.6%) in ovary, and 24,116 (24.6%) in unfertilized eggs (Figure 4a). All detected transcripts in each library were considered as an expressed transcript and, in particular, we defined the transcripts expressed in the unfertilized eggs library as “maternal transcripts”. Functional classification and enrichment analysis of the maternal transcripts revealed a wide distribution of GO terms and KEGG pathways and 27 GO terms and 39 KEGG pathways being enriched (*p* < 0.05; Figure 2 and Figure 3 and Table S2). In detail, most enriched GO terms included the cellular process (1441 transcripts) in the BP category, catalytic activity (989 transcripts) in the MF category, and cell and cell part (1261 transcripts) in the CC category (Figure 2). While, signal transduction (377 KOs), global and overview maps (236 KOs), endocrine system (187 KOs), and immune system (171 KOs) were the most enriched KEGG pathways (Figure 3). These results indicate that various transcripts involved in a wide range of biological functions and pathways were stored during oogenesis, and these transcripts will be contributed in early embryonic developmental competence until the onset of zygotic transcription [46]. The expression profile with expression abundance distribution and pattern revealed that the transcripts expressed in unfertilized eggs were highly correlated with those in the ovary (yolk-laden mature ovary) (Figure 4b,c), reflecting the maternal RNA transcripts are either produced by the oocyte or stored by surrounding cells into the oocyte during oocyte development [2,47,48]. Nevertheless, there were 3248 (3.3%) transcripts that were only detected in the unfertilized eggs library (Figure 4a). Recent transcriptome studies of zebrafish follicles using serial analysis of the gene expression (SAGE) method provided an extensive data set on maternal mRNA stored in follicles near the end of oogenesis [49,50]. Consistently, in the context of the understanding of ovary development and oogenesis, the transcripts detected only in unfertilized eggs suggests that a number of transcripts were stored in unfertilized eggs near the end of ovarian development and before ovulation of *M. anguillicaudatus*. Meanwhile, there were 38,163 (38.9%) transcripts were coexpressed in more than two tissues, of which 20,868 (86.5%) transcripts were coexpressed between unfertilized eggs and other tissues (Figure 4a).

Pleiotropy is a common phenomenon caused by a single molecular function involved in multiple biological processes, which could affect two or more distinct and seemingly unrelated traits [51,52,53]. A previous study showed that a number of maternally loaded RNA transcripts in unfertilized eggs were linked to male function in *Tribolium castaneum*, suggesting a pleiotropic function of maternal transcripts [53]. Moreover, high-through RNA interference data from *Caenorhabditis elegans* has shown that pleiotropic genes were involved in an important role in early embryological life and functioned as a potential bridge between different protein complexes and pathways [54]. In this respect, we believed that function of maternal transcripts can be deduced from their tissue-specific coexpressed transcripts of known function [55,56], and therefore, we further classified and characterized transcripts that were coexpressed in the unfertilized eggs with other tissues using the TissueEnrich tool [57]. Among maternal transcripts coexpressed in other tissues, 2806, 4384, 5349, and 6281 were grouped into specific transcripts (tissue enriched and group enriched transcripts) with muscle, testis, ovary, and unfertilized eggs, respectively, while 6050 and 5358 transcripts were classified into expressed in all and mixed group (Figure 4d and Table S4). The GO annotation by WEGO revealed that a number of the maternal transcripts linked with tissue specificity was significantly different in several GO terms (*p* < 0.05; Figure 4e). As such, it had been found that not only large numbers of the maternal transcript were linked to functions with ovarian specificity, but large numbers of the maternal transcripts were also linked to functions with testicular and muscle specificity, implying that these transcripts would have a pleiotropic function linked with tissue-specificity and affect multiple phenotypic traits. Surprisingly, we found that, in each tissue-specific coexpressed transcript, highly expressed transcripts (TMM value > 50) with high percentages (99.1% muscle-, 86.2% testis-, and 94.8% ovary-specific) appeared in maternal transcripts (Figure 4d). To date, many studies have made an effort to identify genes/transcripts associated with egg quality as the ability of the egg to be fertilized and subsequently develop into a normal embryo [8]. Nevertheless, the information of genes and biological mechanisms as a determinant of egg quality is still poorly understood and there is no study of maternal transcripts associated with the proportion and abundance of tissue-specific transcripts. Although there are many biological questions and debates about the proportion of maternal transcripts linking with tissue-specificity, it could be valuable to examine potential interrelationship between contents of tissue-specific material transcripts and indices of egg quality.

### 2.4. Identification of Maternal Gene Transcripts Related to Immune Functions

**Figure 5:**
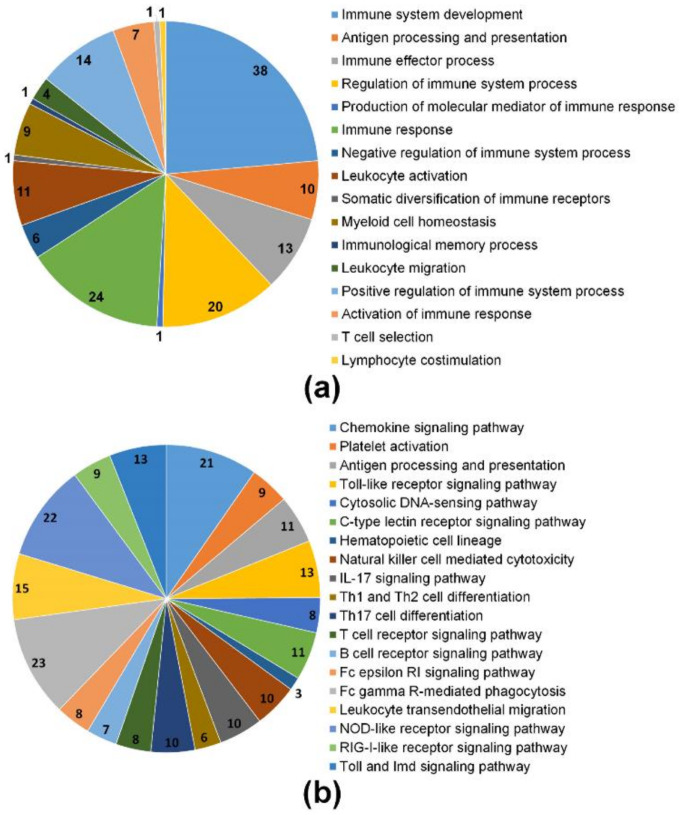
Identification of maternal immune-related gene transcripts based on GO and KEGG pathway annotations: (**a**) Distribution of maternal gene transcripts in the immune system process (GO:0002367) at GO level 3 and (**b**) distribution of maternal gene transcripts in the immune system pathway subcategories.

To further investigate maternal transcripts associated with immune function during early embryo development in *M. anguillicaudatus*, which may also have a pleiotropic function as mentioned above or share the function to the developmental mechanism in early embryonic development, we identified candidate transcripts related to immune functions. We focused on the GO term of immune system process (GO:0002367) and the KEGG pathway of immune system (5.1), which was closely associated with immune functions, processes, and interactions and often used to identify genes/transcripts associated with the immune functions. A total of 61 maternal transcripts were classified into 16 different GO terms (level 3) in the GO immune system process and 218 maternal transcripts with 171 KOs were assigned into 19 signaling pathways in the KEGG immune system (Figure 5). The top three GO terms that most frequently represent maternal gene transcripts in the immune system process are the immune system development (GO:0002520, 38 transcripts from 33 genes), the immune response (GO:0006955, 24 transcripts from 15 genes), and the regulation of the immune system process (GO:0002682, 20 transcripts from 14 genes; Figure 5a). Moreover, in the immune system KEGG pathway, the three pathways with the largest number of maternal gene transcripts were the chemokine signaling pathway (21 maternal transcripts with 16 KOs), the Fc gamma R-mediated phagocytosis (23 transcripts with 12 KOs), and the leukocyte transendothelial migration (15 transcripts with 12 KOs; Figure 5b). Among them, ten maternal gene transcripts were involved in multiple immunological systems with high expression (TMM value > 50), which seem to correlate with diverse cellular functions during early embryonic development of *M. anguillicaudatus* (Table 2). Detailed information on the maternal transcripts involved in immunological functions is shown in Table S5. Overall, our results indicate that a variety of maternal immune-related gene transcripts were stored during oocyte development, which can facilitate effective defense mechanisms against invading pathogens during embryonic development before the maturation of immunocompetence in *M. anguillicaudatus* embryos.

### 2.5. Transcriptional Levels of Maternal Immune-Related Candidate Genes during Development and Early Ontogeny of M. anguillicaudatus

Seven maternal genes involved in multiple immunological systems with high expression (TMM value > 50) were further studied with qPCR analysis. They included genes, *RAC1*, *MAPK3*, *MAPK14*, *IL1B*, *CTNB1*, *FADD*, and *ITB1*. Preferentially, we validated the expression profile of the selected candidate genes from RNA-Seq analysis using qPCR. The qPCR results revealed that expression of the candidate genes was consistent with expression profiles from RNA-Seq analysis (Figure S2), indicating that our RNA-Seq data was accurate and effective and that expression profiles of maternal gene transcripts can be used for further studies, such as ontogenetic expression where the adaptive immune system is still far from being completely developed.

**Figure 6:**
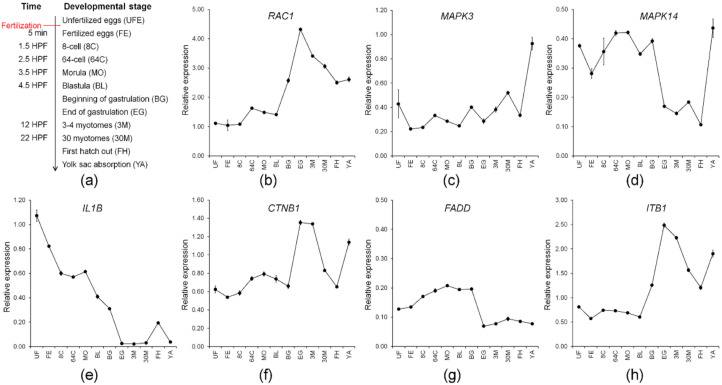
Transcriptional levels of identified maternal immune-related candidate genes during ontogeny of *M. anguillicaudatus*: (**a**) the ontogeny of *M. anguillicaudatus*, time for each developmental stage was represented by HPF (hours post-fertilization) and DPH (days post-hatching) and transcriptional levels of (**b**) *RAC1*; (**c**) *MAPK3*; (**d**) *MAPK14*; (**e**) *IL1B*; (**f**) *CTNB1*; (**g**) *FADD*; and (**h**) *ITB1*. The results analyzed in statistics are shown in Table S6.

A study on zebrafish miR430 has suggested that a correctly timed degradation of maternal transcripts is critical for embryogenesis to transfer developmental control from the maternal transcripts to the zygotic genome [58]. In this context, evaluation of transcriptional levels of maternal candidate genes can provide an understanding of the immune system and embryogenesis involvement of maternal immune-related genes during embryogenesis and larval ontogeny. Consequently, we evaluated the transcription levels of identified maternal immune-related candidate genes during the development of *M. anguillicaudatus* (Figure 6a). Expression of all maternal immune-related candidate genes, except for *FADD*, exhibited a drastic increase after first hatching out, which has an agreement that development of the immune function starts after hatching in *M. anguillicaudatus* (Figure 6) [59]. The expression patterns of *RAC1*, *CTNB1*, and *ITB1* were similar, which the transcripts retained until the blastula stage, then began to increase at the beginning of gastrulation, and peaked at the end of gastrulation (Figure 6b,f,h). Consistent with this, expression of *RAC1* mRNA in zebrafish was found in 30% epiboly that occurred during the blastula period after the yolk syncytial layer forms (YSL) [60] and the zygotic expression of *RAC1* mRNA clearly showed the involvement in the host immune response by microbial infection in fish [60,61,62]. *CTNB1* is well-known as a maternal gene, which mediates the canonical Wnt signaling pathway and plays a crucial role in the generation of the body plan in zebrafish embryo [63,64]. Thus, the result indicated that *RAC1*, *CTNB1*, and *ITB1* seem to be “typical” for maternal transcripts that could be degraded during midblastula transition (MBT). These transcripts are responsible for defense against pathogen invasion and/or embryonic development of *M. anguillicaudatus*. MAPKs generally are known as a protein kinase group and are involved in directing a diverse array of cellular functions including proliferation, gene expression, differentiation, mitosis, and apoptosis. A knock-down experiment in zebrafish showed *MAPK3* and *MAPK14* mRNA expression was required at an early embryonic stage (from the 2,4 cell stage to 50% epiboly) in the gilthead sea bream (*Sparus aurata*) [65,66]. Zygotic expression of *MAPK3* and *MAPK14* were upregulated by the immune challenge in several fish species [65,67]. Expression of maternal *MAPK14* in *M. anguillicaudatus* declined around the gastrulation stage. Meanwhile, the expression pattern of *MAPK3* was steady, so we were unable to determine maternal to zygotic transition, suggesting the expression of *MAPK3*, whether maternal or zygotic expression, is required above a certain transcriptional level for immune function and embryonic development (Figure 6c,d). Studies on IL1B functions involved in the control of inflammatory responses have been extensively conducted in several fish species [68,69,70,71] and maternal expression of *IL1B* was evident in rohu (*Labeo rohita*) and brown trout (*Salmo trutta*) [72,73]. Expression of maternal *IL1B* in *M. anguillicaudatus* gradually declined until the end of gastrulation. Furthermore, there was no change during the myotome stages (Figure 6e). This indicates the expression of maternal *IL1B* is required for early embryonic development rather than the myotomes stage. FADD is an adaptor protein to form the death-inducing signaling complex (DISC) during apoptosis [74]. The function of the FADD gene as a maternal gene is unknown, but the *FADD* knockout mouse model demonstrated that the absence of *FADD* is lethal [75]. We did not observe changes in maternal *FADD* during our study (Figure 6g). Many maternal immune-related genes seem to correlate with diverse cellular functions during early embryonic development. However, how these genes function in early immune development is unclear. Further studies on the role of these maternal gene transcripts in early embryonic and immune system development in *M. anguillicaudatus* and other fish need to be conducted.

### 2.6. Data Availability

The cleaned short read sequences from the RNA-Seq data were deposited in the NCBI Sequence Read Archive (SRA, http://www.ncbi.nlm.nih.gov/sra) under the accession numbers SRX4452604, SRX4452605, SRX4452606, and SRX4452607 for ovary, testis, muscle, and unfertilized eggs, respectively. The Transcriptome Shotgun Assembly (TSA) project has been deposited in the DDBJ/EMBL/GenBank under the accession GGUH00000000. The version described in this paper is the first version, GGUH01000000.

## 3. Materials and Methods

### 3.1. Fish Rearing and Sample Collection

The cyprinid loach, *Misgurnus anguillicaudatus,* used in this study was a laboratory stock maintained at the Institute of Marine Living Modified Organism, Pukyong National University, Busan, Korea. Fishes were reared in water recirculating aquarium tanks filled with sand-filtered groundwater at 25 ± 1 °C and continuous aeration (dissolved oxygen level = 5 ± 1 mg/L). Fishes were fed with a commercial diet (50% crude protein) on an ad libitum basis. For tissue sampling, 2-year-old mature adult fishes were euthanized with tricaine methanesulfonate (MS-222; 500 mg/L). Gonads (yolk-laden mature ovary or milky white-colored testis) were surgically dissected from 3 females (average body weight (BW) = 24.3 ± 3.5 g) and 3 males (average BW = 12.3 ± 1.4 g). In addition, muscles (dorsal skeletal muscles) were obtained from the same individuals above in order to increase transcriptome coverage of the gene expressed in fertilized eggs during de novo transcriptome assembly. Collected samples were immediately frozen on dry ice and stored at −80 °C until RNA extraction. Unfertilized eggs were obtained by hormone-induced spawning, as described previously [27]. Mature females (*n* = 3; same-aged and similar-weighed as above) were given an intraperitoneal injection of carp pituitary extract (Argent Aquaculture Inc., Redmond, WA, USA) at a dose level of 10 μg/g body weight. Thirteen hours after injection at 25 °C, eggs were hand stripped and directly subjected to RNA extraction. This study was approved by the Animal Ethics Committee of Pukyong National University (Approval #2018-16; approval date: 30 Aug. 2018) and performed according to the guidelines for the care and use of laboratory animals.

### 3.2. RNA Isolation, Library Construction, and Sequencing

Total RNAs were extracted from individual tissue and egg batch samples using the Trizol reagent (Invitrogen, CA, USA) and purified using the RNeasy Plus Mini Kit (Qiagen, Hilden, Germany) according to the manufacturer’s instruction, including a genomic DNA elimination step. RNA purity, quality, and quantity were determined by NanoDrop-2000 spectrophotometer (Thermo Scientific, Waltham, MA, USA), ethidium bromide staining of 28S and 18S ribosomal bands on a 1% MOPS-formaldehyde agarose gel, and Bioanalyzer 2100 system (Agilent Technologies Inc., Stanta Clara, CA, USA). Only high-quality RNAs (A260/A280 ratio = 1.8–2.0 and A260/A230 ratio ≥ 2.0, and RIN > 8) were pooled within a given sample type (1 μg of total RNA each from individual sample) to construct cDNA libraries. Briefly, 1 μg of total RNA was purified, fragmented, and primed to synthesize the first- and second-strand cDNA according to the TruSeq Stranded Total RNA Sample Pre protocol (Illumina, San Diego, CA, USA). Synthesized second-strand cDNA was purified, end-repaired, and ligated to index adapters. The ligated products were separated to select suitable sequencing templates that were amplified by PCR to generate cDNA libraries. Finally, the libraries were sequenced using the Illumina HiSeq 2500 (Illumina, Inc., San Diego, CA, USA) platform as paired-end reads to 100 bp in length.

### 3.3. De Novo Assembly and Completeness of Transcriptome

After sequencing was completed, the Trimmomatic v0.32 software was used to process Illumina paired-end raw reads to obtain clean reads through trimming the adaptor sequences, plus trimming of sequences with an ambiguous base (N) ratio >5% and low-quality [30]. Subsequently, the integrity of the remaining raw reads was verified with FastQC v0.10.1 by running Perl program [76] with the clean reads stored in the FASTQ format [77]. In the absence of a reference genome for the loach *M. anguillicaudatus* in this study, the clean reads were pooled together to maximize the chance of obtaining as many transcripts as was possible, and de novo assembly used to create a reference transcriptome using Trinity Assembler v2.2.0 with default parameters [31]. The assembly was clustered by removing the redundant sequences using a cluster software, CD-HITest v4.6.1, with a defined parameter (-c 0.99 -T 8 -G 0 -aL 0.90 -AL 100 -aS 0.99 -AS 30) [32]. Completeness of the de novo transcriptome assembly, as a reference for downstream analysis, was assessed using Benchmarking Universal Single-Copy Orthologs v3.0.2 software (BUSCO, http://busco.ezlab.org/) against the eukaryote (eukaryote_odb9) lineages with default parameters in the CyVerse Discovery Environment (https://de.cyverse.org/) [38,39,78].

### 3.4. Functional Annotation

Initially, transcripts were annotated by homology search using BLASTx algorithm against NCBI NR protein database. In addition, Trinotate v3.0.1 (https://trinotate.github.io/) pipeline was used to search for any additional homologies of the transcripts: the open reading frames (ORF) for each transcript were predicted by TransDecoder v3.0.1 (https://github.com/TransDecoder) [79]. Nucleotide sequences and the corresponding ORF proteins were queried against the Swiss-Prot/UniprotKB database. Protein domains were identified using HMMER v.3.1 against the HMMER/Pfam protein database [80]. Orthologous groups were searched against the EggNOG database [81]. Furthermore, prediction for the presence of the signal peptide and transmembrane regions were performed using SignalP v4.1 [82] and tmHMM v2.0c [83]. The NR BLAST results were imported to the Blast2GO v3.0.11 program for mapping and retrieving gene ontology (GO) [41]. After retrieving the GO annotation result, the WEGO 2.0 tool was used for classification and graphical representation of GO terms at the macro level [42]. GO terms with a corrected *p*-value < 0.05 were defined as significantly enriched GO terms. Kyoto Encyclopedia of Genes and Genomes (KEGG) pathway assignments were performed on the KEGG Automatic Annotation Server (KAAS) [44]. KEGG annotations were filtered to keep KEGG orthology (KO) and those were categorized using the KEGG Mapper (https://www.kegg.jp/kegg/tool/map_pathway.html). Enrichment analysis was conducted by comparison of a transcript set against the reference transcriptome background using the Pearson Chi-Square test in R package. GO terms and KEGG pathways with a *p*-value less than 0.05 were considered to be enriched significantly.

### 3.5. Expression Analysis and Specificity Classification

Expression levels of transcripts from four tissue libraries (muscle, ovary, testis, and unfertilized eggs) were calculated by mapping to the reference transcriptome that was created by the de novo assembly mentioned above. To obtain the expression levels of each transcript in each tissue, the transcript abundances were quantified for each library using Kallisto v0.43.0 [84]. Low abundance transcripts with less than two count across four tissue libraries were removed. To normalize the expression of transcripts across tissues, the trimmed mean normalization of M-values (TMM) was calculated using the Bioconductor package edgeR v3.8.5 [85] and used to classify tissue specificity of transcripts. A cut-off value of 1 TMM was used as the detection limit [86]. To determine the distribution of tissue specificity, transcripts were further classified into six categories using the TissueEnrich tool [57]: (1) “Not expressed”—< 1 TMM value across all the tissues; (2) “Tissue enriched”—five-fold higher TMM value in one tissue compared to all other tissues; (3) “Group enriched”—five-fold higher TMM value in a group of 2-3 tissues compared to all other tissues; (4) five-fold higher TMM value in one tissue compared to the average TMM value in all other tissues; (5) “Expressed all”—1 ≥ TMM value across all of the tissues; and (6) “Mixed”—transcripts that were not assigned to any of the five categories. Transcripts from “Tissue enriched”, “Group enriched”, and “Tissue enhanced” groups were classified as tissue-specific transcripts [57]. For the classification of transcripts where a log2-scale of the data was used, pseudo-counts of +1 were added to the data set.

### 3.6. qPCR Analysis and Measurement of Transcriptional Levels of Identified Candidate Genes during Ontogeny of M. anguillicaudatus

Seven genes from the RNA-Seq analysis and 18s ribosomal RNA (*18S rRNA*, accession no. EU120032) as an endogenous control [87] were analyzed by qPCR to validate RNA-Seq data and to evaluate ontogenetic expression profile. For RNA-Seq data validation, the total RNAs from each tissue were prepared from three replicate pools of tissues (three individuals per pool within a given tissue type) independently of the RNA-Seq analysis. To evaluate the transcriptional levels of identified candidate genes during ontogeny, developmental samples at 25 °C were collected from 12 developmental stages (i.e., unfertilized eggs, immediately after fertilization, 8-cells, 64-cells, morula, blastula, beginning of gastrulation, end of gastrulation, 3-4 myotomes, 30 myotomes, first hatch out, and yolk sac absorption) according to the study on ontogeny of *M. anguillicaudatus* that has been previously described [28]. Three independent spawning trials were performed to prepare biological replications and 60 embryos were sampled at each developmental stage from each spawning batch. Total RNAs were extracted and purified according to the RNA isolation described in Section 3.2. Subsequently, cDNA was synthesized from 2 μg total RNA using the Omniscript reverse transcription kit (Qiagen) with a 1–9 mixing ratio of oligo dT primer and random nonamer primers (Bioneer, Daejeon, Korea), according to the manufacturer’s instruction. RT-qPCR was performed in triplicate using the LightCycler 480 Real-Time PCR system and LightCycler 480 SYBR Green I Master (Roche Applied Science, Penzberg, Germany). In brief, the amplification was carried out in a 20 μL reaction mixture containing 1 × SYBR green I master, 5 μM forward and reverse primers, and 2 μL of 20 times diluted cDNA template and nuclease-free water. The thermal profile conditions were: 90 °C for 5 min, 40 cycles consisting of 95 °C for 10 s, 60 °C for 20 s, and 72 °C for 20 s, with fluorescence recording at the end of each cycle. Melt curve analysis was performed to ensure product specificity over the temperature range of 65–90 °C. The sequences of oligonucleotide primers used in qPCR are listed in Table S7. Amplicons were analyzed on agarose gels to confirm the product size. The levels of gene expression were calculated from the threshold cycle according to the 2^-ΔΔCT^ method [88]. The results were analyzed one-way analysis of variance (ANOVA) with a Bonferroni’s multiple comparison test using GraphPad Prism (v7.0 for Windows; GraphPad Software). A *p* < 0.05 was considered statistically significant.

## 4. Conclusions

Our study presented the transcriptome expression profiles of the loach, *M. anguillicaudatus*, and for the first time, maternal gene transcripts and their immune system involvement were identified from unfertilized eggs of *M. anguillicaudatus*. These findings are needed to understand maternal gene transcripts in early embryonic development and the immunological understanding of maternal immune-related gene transcripts before zygotic gene expression. This information can be utilized to assess developmental competence concerning cellular mechanism and/or immune system in embryonic development, which can be associated with the egg quality of teleost fish. The data generated could be a valuable transcriptomic/genomic resource for further analysis of maternal genes involved in the cellular mechanisms and/or immune system in early embryonic development of *M. anguillicaudatus* and other teleost fishes.

## Figures and Tables

**Table 1:** Statistics for sequencing, de novo assembly, and completeness assessment of the *M. anguillicaudatus* transcriptome.

**Sequencing Stats** (Illumina HiSeq 2500; paired-end, 2 × 101 bp)					
Sequencing	Ovary	Testis	Muscle	Unfertilized eggs	Total
No. of raw reads	63,490,556	60,095,449	56,211,235	63,470,524	243,267,764
No. of clean reads	62,078,597	58,749,280	55,006,889	62,034,978	237,869,744
Q20 of clean read	100%	100%	100%	100%	100%
***De novo*Assembly Stats** (Trinity v2.2.0)					
No. of total genes	No. of total transcripts	Total size (bp)	Mean size (bp)	N50	GC (%)
202,709	281,866	218,148,238	773.94	1692	42.96
**After Removing Non-Redundant sequzences** (CD-HITest v4.6.1)					
No. of total genes	No. of total transcripts	Total size (bp)	Mean size (bp)	N50	GC (%)
193,289	267,111	197,693,642	740.12	1533	42.90
**Expression Profiling** (Kallisto v0.43.0 and edgeR v3.8.5)					
Description	Ovary	Testis	Muscle	Unfertilized eggs	Average
Mapping ratio (%)	95	92	89	95	92.8
No. of TMM ≥ 1	22,233	73,683	45,603	24,116	

**Table 2:** Maternal genes involved in multiple immune systems with high expression levels (TMM value > 50).

Gene	Description	No. of Involved Immune System	TMM Value
*RAC1*	Ras-related C3 botulinum toxin substrate 1	15	87.41
*MAPK3*	mitogen-activated protein kinase 3	13	60.25
*MAPK14*	mitogen-activated protein kinase 14	12	61.73
*IL1B*	interleukin-1 beta	7	216.87
*CTNB1*	catenin beta-1	5	138.05
*FADD*	FAS-associated death domain protein	5	65.82
*ITB1*	Integrin beta 1	2	166.79
*LEF1*	lymphoid enhancer-binding factor 1	2	61.20
*MELK*	maternal embryonic leucine zipper kinase	2	76.41
*PDPK1*	3-phosphoinositide-dependent protein kinase 1	2	127.54

## Supplementary Materials

Supplementary materials can be found at https://www.mdpi.com/1422-0067/21/11/3872/s1.

## Abbreviations

RIN	RNA integrity number
BUSCO	Benchmarking Universal Single-Copy Orthologs
KEGG	Kyoto Encyclopedia of Genes and Genomes
GO	Gene ontology
TMM	Trimmed mean normalization of M-values
