# Supplementary material for: Transcriptome Analysis of Maternal Gene Transcripts in Unfertilized Eggs of Misgurnus anguillicaudatus and Identification of Immune-Related Maternal Genes
Source: Int J Mol Sci. 2020 May 29;21(11):3872. doi: 10.3390/ijms21113872 (PMC7312655; doi:10.3390/ijms21113872)
Supplement: Supplementary file 1 [file ijms-21-03872-s001.zip › Figure S2.docx]

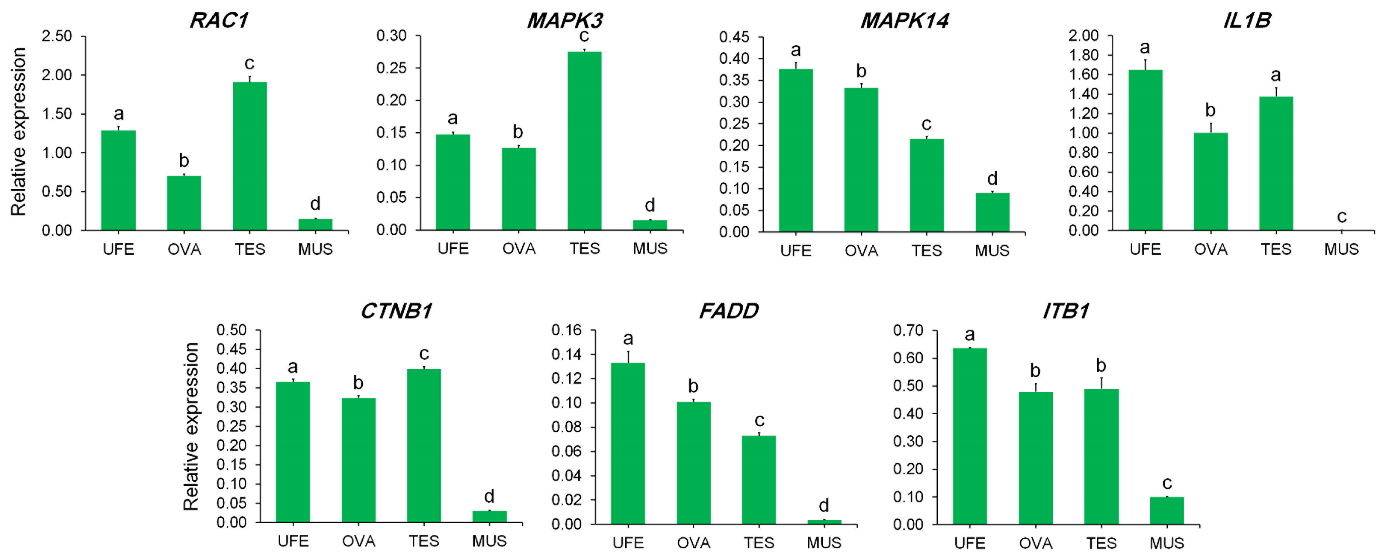


**Figure S2.** qPCR validation of candidate maternal transcripts related to immune system, which were identified from RNA-Seq analysis. Mean ± standard deviation (n = 3) are shown. Means denoted by the different letters (a, b, c, and d) at the top of the bars indicate statistically significant differences (*p* < 0.05) between tissues determined by one-way ANOVA followed by Bonferroni's multiple comparison test. UFE (unfertilized eggs), OVA (ovary), TES (testis), and MUS (muscle).
