# Supplementary figures and images for: Transcriptome Analysis of Maternal Gene Transcripts in Unfertilized Eggs of Misgurnus anguillicaudatus and Identification of Immune-Related Maternal Genes
Source: Int J Mol Sci. 2020 May 29;21(11):3872. doi: 10.3390/ijms21113872 (PMC7312655; doi:10.3390/ijms21113872)

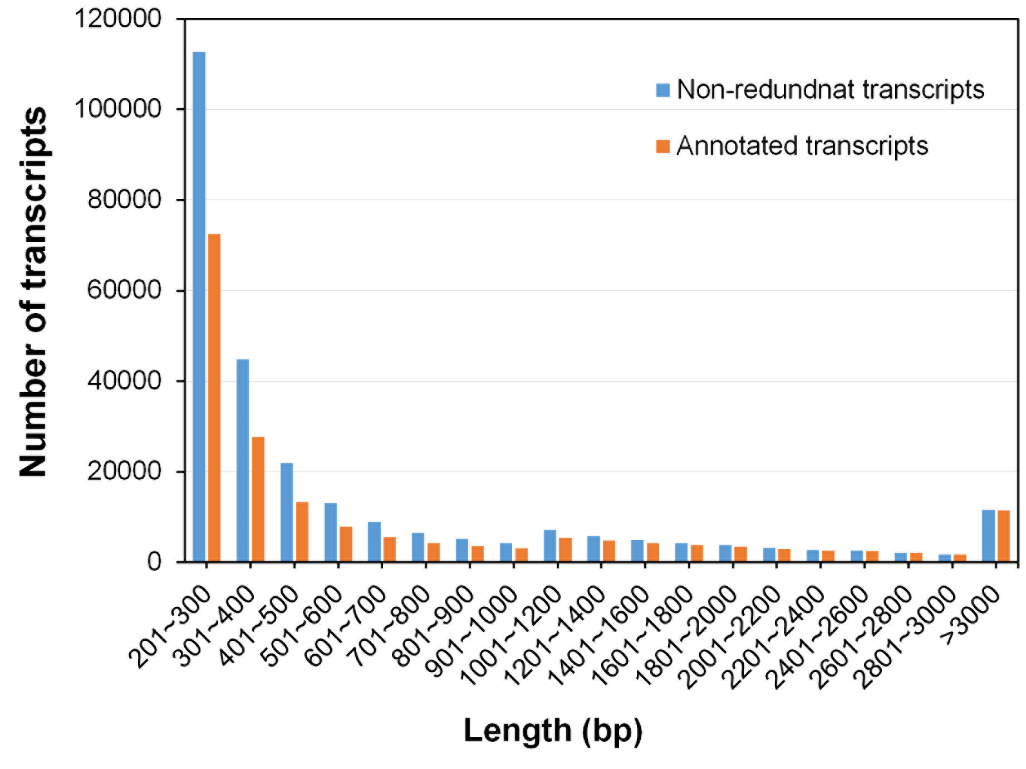


**Figure S1.** Length distribution of all non-redundant transcripts and annotated transcripts.

Supplement: Supplementary file 1 [file ijms-21-03872-s001.zip › Figure S1.docx]
